# Incidental Cardiac Uptake on ^99m^Tc-HMDP Bone Scintigraphy in Oncology Patients: Two Cases of Transthyretin Amyloid Cardiomyopathy with Literature Review

**DOI:** 10.3390/diseases14010023

**Published:** 2026-01-07

**Authors:** Naoya Matsuki, Toru Awaya, Jin Endo, Taeko Kunimasa, Tatsuya Gomi, Yasushi Okamoto, Hidehiko Hara

**Affiliations:** 1 Division of Radiology, Toho University Ohashi Medical Center, 2-22-36, Ohashi Meguro-ku, Tokyo 153-8515, Japan; naoya.matsuki@med.toho-u.ac.jp; 2 Department of Cardiovascular Medicine, Toho University Ohashi Medical Center, 2-22-36, Ohashi Meguro-ku, Tokyo 153-8515, Japan; 3 Department of Cardiology, Keio University School of Medicine, 35 Shinanomachi, Shinjuku-ku, Tokyo 160-8582, Japan; 4 Department of Radiology, Toho University Ohashi Medical Center, 2-22-36, Ohashi Meguro-ku, Tokyo 153-8515, Japan; 5 Department of Breast Surgery, Toho University Ohashi Medical Center, 2-22-36, Ohashi Meguro-ku, Tokyo 153-8515, Japan

**Keywords:** bone metastasis, transthyretin amyloid cardiomyopathy, ^99m^Tc-hydroxymethylene diphosphonate (HMDP), bone scintigraphy, cerebral infarction, oncology

## Abstract

Background: Bone scintigraphy using technetium-99m hydroxymethylene diphosphonate (^99m^Tc-HMDP) is extensively employed to detect bone metastases. However, incidental myocardial uptake may indicate wild-type transthyretin amyloid cardiomyopathy (ATTRwt-CM), a frequently overlooked diagnosis with important clinical implications. Case Presentation: Two elderly female patients with a history of breast cancer were subjected to ^99m^Tc-HMDP bone scintigraphy as part of a routine evaluation for possible bone metastases. Both cases demonstrated incidental myocardial uptake (Perugini Grade 2 and Grade 3, respectively), raising suspicion for ATTRwt-CM, which was subsequently confirmed by endomyocardial biopsy. Review of the Literature: We reviewed published studies reporting cardiac uptake on bone scintigraphy, summarizing the frequency, patient demographics, and tracer types, and emphasizing the clinical relevance of this finding in cancer patients. Conclusions: In oncology patients, bone scintigraphy performed during routine metastatic screening may facilitate early detection of ATTRwt-CM, enabling timely diagnosis and treatment initiation, potentially improving clinical outcomes.

## 1. Introduction

Wild-type transthyretin amyloid cardiomyopathy (ATTRwt-CM) is a progressive disease caused by the deposition of amyloid fibrils in the myocardium, with increased prevalence in the elderly population [1]. Bone scintigraphy has been established as a non-invasive method for evaluating amyloid fibril deposition [2]. Technetium-99m-labeled bone tracers, including pyrophosphate (^99m^Tc-PYP), hydroxymethylene diphosphonate (^99m^Tc-HMDP), and 3,3-diphosphono-1,2-propanodicarboxylic acid (^99m^Tc-DPD), are used to detect cardiac amyloidosis [3]. Notably, ^99m^Tc-HMDP and ^99m^Tc-DPD are also commonly used for assessing bone metastases in cancer patients [4]. In cancer survivors undergoing routine bone scintigraphy, incidental cardiac uptake is sometimes observed (3.6%), which may indicate underlying cardiac amyloidosis [5].

Survivors of breast and prostate cancer are known to be at increased risk of cardiovascular events, including heart failure, due to prior exposure to cardiotoxic chemotherapy or hormone therapy, as well as other contributing factors [6,7]. Therefore, in this patient population, careful attention to incidental cardiac uptake on bone scintigraphy may facilitate early diagnosis of transthyretin amyloid cardiomyopathy (ATTR-CM) and help prevent heart failure.

Cardiac uptake on bone scintigraphy is assessed using the Perugini grading system, with a classification of Grade 0 (no cardiac uptake), Grade 1 (less than bone), Grade 2 (equal to bone), and Grade 3 (greater than bone). The heart-to-contralateral (H/CL) ratio is also used for quantitative assessment, with a ratio > 1.3 at 3 h considered positive. Grade 2 and 3 uptake on bone scintigraphy may indicate ATTR-CM [8], with Grade 3 uptake particularly identified as an independent predictor of the overall and cardiovascular mortality [5].

In light of the steady development of ATTR-CM therapies, which include both wild and variant types, early recognition is becoming more critical [9,10], and attention to incidental cardiac uptake on bone scintigraphy performed for metastatic evaluation is warranted, particularly in elderly cancer survivors.

In this review, we present two cases of incidental cardiac uptake detected on ^99m^Tc-HMDP bone scintigraphy in breast cancer patients and provide a literature review to highlight its clinical relevance and the need for increased awareness of ATTRwt-CM in oncology patients.

## 2. Aim and Methods of the Present Review

**Aim.** To contextualize the present two cases of incidental cardiac uptake on ^99m^Tc-HMDP bone scintigraphy within the existing evidence in terms of the prevalence, clinical significance, and diagnostic implications of myocardial tracer uptake in oncology populations. Specifically, we sought to evaluate how incidental uptake identified during routine metastatic screening could facilitate early identification of ATTRwt-CM in elderly cancer survivors.

**Search Strategy and Study Selection.** A narrative literature review was conducted using PubMed and Google Scholar from inception until May 2025. Search terms included “bone scintigraphy,” “HMDP,” “DPD,” “HDP,” “PYP,” “cardiac uptake,” “transthyretin amyloidosis,” “ATTR,” “incidental,” “oncology,” and “cancer”. Studies were included if they (1) reported myocardial uptake on ^99m^Tc-labeled bone scintigraphy (HMDP, DPD, HDP, or PYP), and (2) involved either oncology patients or mixed populations from which oncology-specific data could be extracted. Cohort studies, case series, and individual case reports were all eligible. While studies using ^99m^Tc-MDP were excluded from the primary synthesis due to its low sensitivity for ATTR-CM detection, MDP-based cases with biopsy-confirmed amyloidosis were included in a separate table for completeness.

**Scope of the Review.** This review offers an overview of (a) the reported prevalence of incidental cardiac uptake in oncology and general populations, (b) tracer-specific diagnostic considerations, (c) potential interpretative pitfalls, and (d) clinical outcomes, including thromboembolic complications. By integrating evidence from recent studies with the present two cases, we aim to demonstrate the clinical importance of recognizing incidental myocardial uptake in routine bone scintigraphy and its significant role in the early detection of ATTRwt-CM among elderly cancer survivors.

## 3. Case Report

### 3.1. Case 1

**Figure 1:**
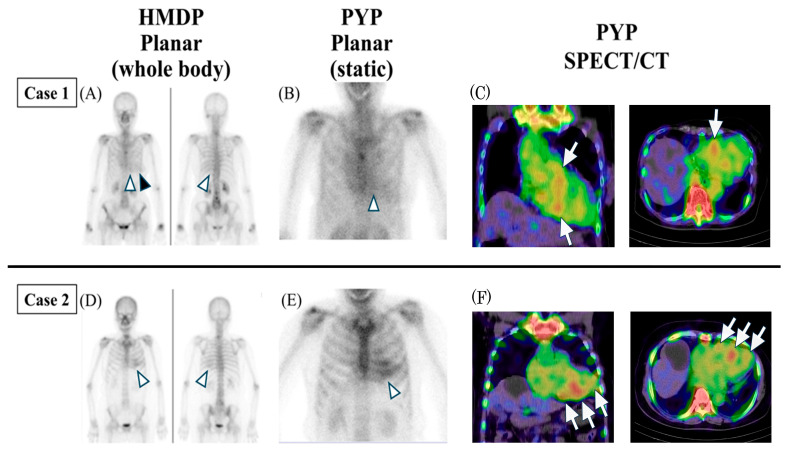
Incidental ATTRwt-CM on Bone Scintigraphy in Oncology Patients. **Case 1**: (**A**) ^99m^Tc-HMDP scintigraphy (3 h post-injection) revealed cardiac uptake near the sternum (Perugini Grade 2) (arrowhead). However, evaluation was limited by tracer uptake in the blood pool and breast tissue (black arrowhead). (**B**) ^99m^Tc-PYP planar image showed equivocal uptake (H/CL ratio 1.4; normal <1.3 at 3 h) (white arrowhead). (**C**) ^99m^Tc-PYP SPECT/CT demonstrated suspected myocardial uptake in the basal septum (white arrows), consistent with true myocardial localisation over blood pool and breast tissue uptake. **Case 2**: (**D**) ^99m^Tc-HMDP scintigraphy for bone metastasis screening revealed cardiac uptake (Perugini Grade 3) (arrowhead). (**E**) ^99m^Tc-PYP planar image for cardiac amyloidosis also showed uptake (H/CL ratio 2.2; normal <1.3 at 3 h) (arrowhead). (**F**) ^99m^Tc-PYP SPECT/CT demonstrated suspected myocardial uptake in the biventricular inferior and septal regions (white arrows). ^99m^Tc-HMDP, technetium-99m hydroxymethylene diphosphonate; ^99m^Tc-PYP, technetium-99m pyrophosphate; H/CL, heart-to-contralateral; SPECT/CT, single-photon emission computed tomography/computed tomography; h, hours.

An 84-year-old woman, who had undergone right breast-conserving surgery (stage IA) nine years earlier and showed no evidence of recurrence, underwent bone scintigraphy with ^99m^Tc-HMDP in 20XX to evaluate bone metastases. The results revealed abnormal myocardial uptake, suggesting cardiac amyloidosis (Figure 1A). She had no history of myocardial infarction or structural heart disease, and the myocardial uptake was therefore considered a truly incidental finding. Electrocardiography indicated reversed R-wave progression in the precordial leads. Transthoracic echocardiography demonstrated mild hypertrophy with sigmoid septum and reduced longitudinal strain in the basal interventricular septum. Furthermore, laboratory tests detected elevated high-sensitivity troponin T (hsTnT) (0.021 ng/mL) and N-terminal pro-brain natriuretic peptide (NT-proBNP) (1554 pg/mL). However, the patient exhibited no symptoms of heart failure. Serum free light chains (FLC) were within normal limits, and no monoclonal protein was detected. Although the ^99m^Tc-HMDP image showed Perugini Grade 2 uptake (Figure 1A), myocardial uptake remained inconclusive. Thus, ^99m^Tc-pyrophosphate (PYP) scintigraphy, including single-photon emission computed tomography/computed tomography (SPECT/CT), was performed and confirmed myocardial involvement (Figure 1B,C). For ^99m^Tc-PYP imaging, 740 MBq was administered intravenously, and planar images and SPECT/CT were acquired 3 h post-injection using a Symbia Intevo2 SPECT/CT system (Siemens Healthineers, Erlangen, Germany). SPECT acquisition was performed with a low-energy high-resolution collimator, matrix size of 256 × 256, 30 views, 30 s per view, zoom factor of 1.00, non-circular orbit, and step-and-shoot acquisition mode. Image reconstruction was performed using the Ordered Subset Conjugate Gradient Minimizer method with 36 iterations and 1 subset, including scatter correction, CT-based attenuation correction, and 2D-Gaussian smoothing with a full width at half maximum of 20.0 mm. Moreover, right ventricular endomyocardial biopsy was carried out. Congo red and transthyretin staining displayed limited positivity under light microscopy, and ultrastructural examination by electron microscopy confirmed amyloid fibril deposition. Finally, genetic testing was negative. Based on these findings, the patient was diagnosed with ATTRwt-CM. The patient was subsequently treated with tafamidis, a transthyretin tetramer stabilizer, for ATTRwt-CM treatment.

### 3.2. Case 2

An 80-year-old woman with a history of left breast-conserving surgery (stage IIA) four years prior had been placed under monitoring for chronic atrial fibrillation (AF). In 20XX, the patient underwent follow-up bone scintigraphy with ^99m^Tc-HMDP to assess bone metastases. While metastases were undetected, incidental myocardial uptake was observed (Figure 1D). The ^99m^Tc-HMDP scintigraphy showed Perugini Grade 3 uptake; however, it was unclear whether the uptake was in the rib or in the myocardium. Subsequently, ^99m^Tc-PYP scintigraphy with SPECT/CT was performed, using the same protocol as described in Case 1, showing uptake patterns suggestive of myocardial involvement, although definitive localisation remained uncertain (Figure 1E,F). Therefore, in order to establish a definitive diagnosis, endomyocardial biopsy was planned for histopathological confirmation. Electrocardiography showed AF without other notable abnormalities. Transthoracic echocardiography demonstrated left atrial enlargement without evidence of hypertrophy, while longitudinal strain analysis revealed an apical sparing pattern. Laboratory tests revealed elevated hsTnT (0.018 ng/mL) and NT-proBNP (1383 pg/mL), while serum FLC were within normal range, and monoclonal protein was undetected. Following the ^99m^Tc-PYP scintigraphy, the patient was scheduled for right ventricular endomyocardial biopsy. However, the patient was diagnosed with a cerebral infarction prior to her scheduled hospital admission for endomyocardial biopsy. Although she had been receiving daily anticoagulation therapy for chronic atrial fibrillation with 30 mg of edoxaban (appropriate for her body weight and renal function), left atrial appendage closure (LAAC) was performed to prevent recurrence. The biopsy was conducted concurrently during the same procedure. Subsequently, histopathological analysis revealed transthyretin amyloid deposition. Genetic testing was negative, leading to a diagnosis of ATTRwt-CM. She did not develop neurological sequelae and has been followed without significant disability. After undergoing LAAC and being administered tafamidis for ATTRwt-CM, no recurrence of cerebral infarction was reported during subsequent follow-up.

## 4. Review of Cohort Studies and Case Reports on Incidental Cardiac Uptake for Bone Scintigraphy in Oncology

### 4.1. Diagnostic Role of Bone Scintigraphy in ATTR-CM

This section provides a critical literature review on incidental cardiac uptake in routine bone scintigraphy, stratified into cohort studies and case reports (Table 1 and Table 2), focusing on their prevalence, patient characteristics, and diagnostic implications in oncology populations [5]. Bone scintigraphy represents a key non-invasive diagnosis of ATTR-CM in the ESC guideline. The Perugini grading system is utilized for visual assessment, with Grade 2 or 3 considered diagnostic in the absence of monoclonal protein, supported by echocardiographic or cardiac MRI findings. However, when myocardial localization cannot be confidently confirmed on imaging alone, endomyocardial biopsy should be considered for definitive diagnosis [8]. The mechanism underlying myocardial uptake in ATTR-CM remains unclear, although 99mTc-labeled diphosphonates are believed to bind to microcalcifications and implicate macrophage involvement [11]. ATTR amyloid fibrils contain either full-length TTR (type B) or a mixture of C-terminal fragments and full-length TTR (type A), and the presence of C-terminal fragments has been particularly associated with tracer uptake [12].

**Table 1:** Summary of Cohort Studies on Incidental Cardiac Uptake on Bone Scintigraphy in Overall and Oncology Patients (Excluding ^99m^Tc-MDP Scintigraphy).

Authors [Ref.]	Tracers	Age (Years)	Men	Cardiac Uptake (Grade ≥ 2) in Overall Patients	Oncology Patients	Cardiac Uptake in Oncology Patients
Suomalainen et al. [5]	^99m^Tc-HMDP	78 ± 6	69%	3.5% (69/2000)	90%	3.6% (65/1810)
Halme et al. [13]	^99m^Tc-HMDP	77 ± 10	82%	3.5% (47/1334)	94%	2.9% (36/1253)
Mohamed-Salem et al. [14]	^99m^Tc-HMDP ^99m^Tc-HDP ^99m^Tc-DPD	80 (77–83)	65%	2.8% (31/1114)	95.7%	Not reported
Navarro-Saez et al. [15]	^99m^Tc-DPD	78 (74–84)	51%	2.3% (82/3629)	60%	Not reported
Nitsche et al. [16]	^99m^Tc-DPD	64 (51–73)	37%	1.5% (167/11,527)	73.6%	Not reported
de Haro Del Moral et al. [17]	^99m^Tc-HMDP ^99m^Tc-HDP ^99m^Tc-DPD ^99m^Tc-PYP	83.2 ± 6.1	85.9%	0.55% (54/9864)	91.5%	Not reported
Bianco et al. [18]	^99m^Tc-HDP ^99m^Tc-DPD	83 ± 5	78%	0.54% (23/4228)	47.9%	Not reported
Cuscaden et al. [19] †	^99m^Tc-HMDP	–	53%	0.43% (15/3472)	Not specified	Not reported
Longhi et al. [20]	^99m^Tc-DPD	74 (65–82)	37%	0.36% (45/12,400)	95%	Not reported
Salvalaggio et al. [21] ‡	^99m^Tc-HMDP ^99m^Tc-DPD	79 ± 8	–	0.21% (20/9616)	Not specified	Not reported
Son et al. [22]	^99m^Tc-DPD ^99m^Tc-HMDP	55 ± 17	54%	0.07% (23/32,245)	53%	Not reported
Kim et al. [23] §	^99m^Tc-DPD	–	–	0.06% (6/9580)	Not specified	Not reported

Age: Given as mean ± SD, or median [25th–75th percentiles], as reported in the original articles. † Only the mean age was reported; standard deviation was not provided. The study also analyzed a ^99m^Tc-MDP cohort (these were excluded due to the significantly low sensitivity of ^99m^Tc-MDP for ATTR-CM detection). Age data for the entire cohort were unavailable. ‡ Sex data were reported only for cardiac uptake patients (80% men). § Age and sex data reported only for positive cases (mean 80.7 years). Not available for the full cohort.

Table 1 summarizes data from cohort studies on the frequency of incidental cardiac uptake (Grade ≥ 2) in patients undergoing bone scintigraphy [5,13,14,15,16,18,19,20,21,22,23], including ^99m^Tc-HMDP/DPD/HDP/PYP [3,24]. ^99m^Tc-HMDP is also commonly referred to as ^99m^Tc-HDP.

**Table 2:** Summary of Case Reports on Incidental Cardiac Uptake on Bone Scintigraphy in Oncology (Including Confirmed Cardiac Amyloidosis with ^99m^Tc-MDP Scintigraphy).

Authors [Ref.]	Tracers	Age (Years)	Gender	Oncology Subtype	Amyloid Subtype
Tanaka H et al. [25]	^99m^Tc-HMDP	73	Men	Lung cancer	ATTRwt
Delaney et al. [26]	^99m^Tc-DPD	77	Men	Prostate cancer	ATTR
Chono T et al. [27]	^99m^Tc-HMDP	70s	Men	Prostate cancer	ATTR
Lu Y et al. [28]	^99m^Tc-MDP	68	Men	Prostate cancer	ATTR
Fathala A. [29]	^99m^Tc-MDP	86	Men	Prostate cancer	ATTR
Lin et al. [30]	^99m^Tc-MDP	63	Men	Lung cancer	AL
Ikebe et al. [31]	^99m^Tc-HMDP	83	Men	Prostate cancer	ATTRwt

Table 2 presents case reports in which incidental cardiac uptake on bone scintigraphy in oncology patients was followed by a confirmed diagnosis of cardiac amyloidosis. In contrast to Table 1, cases using ^99m^Tc-MDP were also included here if the diagnosis of amyloidosis was subsequently confirmed according to appropriate diagnostic criteria. Most reported cases were of prostate cancer, predominantly ATTR [25,26,27,28,29].

### 4.2. Prevalence of Incidental Cardiac Uptake on Bone Scintigraphy

Cardiac uptake frequency ranged from 0.06% to 3.5% in the general population (Table 1) [5,13,14,15,16,18,19,20,21,22,23]. Notably, most scintigraphy studies showing cardiac uptake were conducted for non-cardiac indications—mainly cancer—with fewer examinations for rheumatological conditions or trauma, and only a small minority for suspected cardiomyopathy. In the study by Nitsche et al., approximately 11% of patients evaluated for suspected cardiac disease demonstrated cardiac amyloid uptake (Perugini grade ≥ 2), whereas only 0.4% of those undergoing bone scintigraphy for non-cardiac purposes, most commonly cancer, showed uptake [16]. However, even in non-cardiac settings, clinical features such as advanced age, male sex, atrial fibrillation, and carpal tunnel syndrome can elevate the likelihood of cardiac uptake and warrant careful consideration of underlying amyloidosis [15]. For instance, a representative cohort study reported uptake rates as high as 6.2% in men and 1.7% in women aged 85 years or older [19].

**Figure 2:**
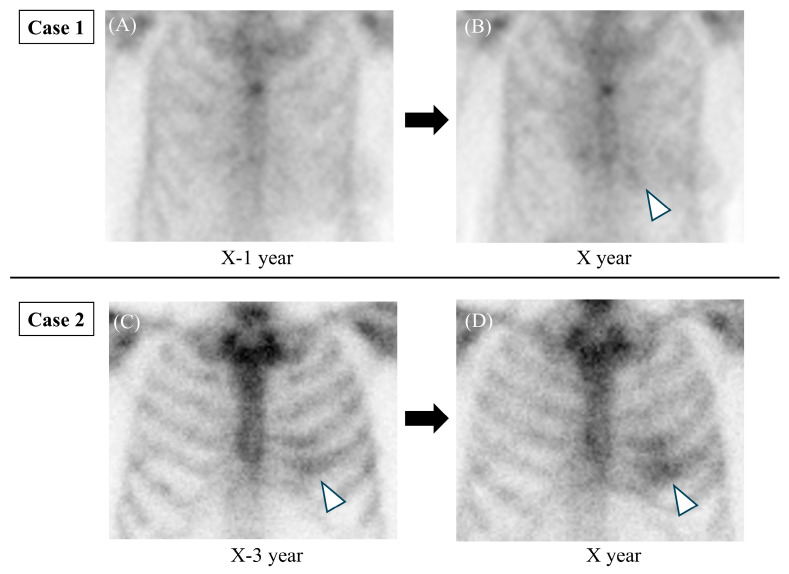
Serial bone scintigraphy during metastatic evaluation in breast cancer patients. Case 1: (**A**) Cardiac uptake was absent in year X−1 (Perugini Grade 0), whereas (**B**) uptake appeared in year X (Perugini Grade 2) (arrowhead). Case 2: (**C**) Cardiac uptake was not evident in year X−3, although retrospective review suggests the possibility of equivocal uptake (Perugini Grade 2) (arrowhead). (**D**) In year X, cardiac uptake was clearly observed (Perugini Grade 3) (arrowhead).

Moreover, incidental cardiac uptake on bone scintigraphy suggested asymptomatic cardiac amyloidosis in 21.7% of patients prior to the development of arrhythmias or heart failure [18], while approximately 60% of patients with cardiac uptake had no heart failure symptoms [5]. Additionally, among ATTR-CM patients who experienced cerebral ischemic events, 16.7% had these events before diagnosis [32]. Together, these findings highlight the potential role of bone scintigraphy in early detection of ATTR-CM, as illustrated in Case 1 and Case 2 (Figure 1 and Figure 2)

### 4.3. Prevalence and Predictors of Incidental Cardiac Uptake in Cancer Patients

Suomalainen et al. reported ATTR-positive cardiac uptake (Perugini grade ≥ 2) in 16 of 384 breast cancer patients (4.2%), compared with 3 of 159 patients (1.9%) in Halme et al.’s findings. For prostate cancer, Suomalainen et al. observed ATTR-positive cardiac uptake in 49 of 1426 patients (3.4%), while Halme et al. reported positivity in 31 of 1013 patients (3.1%) [5,13] (Table 1). In the study by Suomalainen et al., in which approximately 90% of the participants were oncology patients, bone metastasis, an elevated heart-to-contralateral (H/CL) ratio, and Perugini Grade 3 uptake were independent predictors of both overall and cardiovascular mortality [5]. Most studies included mixed populations, and few clearly reported uptake frequency specifically in cancer patients, highlighting the need for further investigation in this subgroup.

### 4.4. Diagnostic Limitations of ^99m^Tc-MDP for ATTR-CM

To maintain diagnostic consistency, studies using 99mTc-methylene diphosphonate (^99m^Tc-MDP) were excluded due to its low sensitivity in ATTR-CM detection [19,33,34], despite its extensive application as a vital radiotracer for bone scintigraphy [4]. Current cardiac amyloidosis guidelines specify the use of ^99m^Tc-PYP/DPD/HMDP/HDP and, appropriately, do not include ^99m^Tc-MDP [3]. Cuscaden et al. [19] reported cardiac uptake in 15 out of 3472 ^99m^Tc-HMDP scans, in contrast to only 1 out of 3446 ^99m^Tc-MDP scans demonstrating positive uptake. Previous reports have also revealed that even in the same patients, ^99m^Tc-MDP consistently exhibits lower cardiac uptake compared with ^99m^Tc-HDP or ^99m^Tc-PYP [34,35]. However, serial ^99m^Tc-MDP scintigraphy has shown progressive cardiac uptake, supporting the diagnosis of cardiac amyloidosis [30]. The lower ^99m^Tc-MDP uptake reflects potential tracer-specific variations in myocardial binding [19,36].

## 5. Serial Bone Scintigraphy in Oncology Patients: Early Signs of Cardiac Amyloidosis and Diagnostic Pitfalls

Serial changes in incidental cardiac uptake during follow-up bone scintigraphy for metastasis are rarely reported (Figure 2) [26,30]. In Case 1, no cardiac uptake was observed in year X−1, whereas uptake appeared in year X, progressing from Perugini Grade 0 to Grade 2 (Figure 2A,B). These serial increases in myocardial uptake observed in this case align with the known progression pattern of cardiac amyloid deposition [2]. Myocardial uptake typically initiates in the basal septum and progressively involves the apical and lateral walls of the left ventricle [37]. Similarly, the right ventricular uptake tends to be more pronounced in the basal segments [38]. Therefore, early uptake near the sternum may be subtle or faint due to superimposed bone structures (Figure 1A–C). This highlights a potential interpretative pitfall. Thus, careful assessment of the parasternal region is warranted to avoid overlooking early signs of cardiac amyloid deposition.

Physiological breast uptake on bone scintigraphy has previously been reported in healthy individuals, particularly in premenopausal women, and is thought to be influenced by factors such as estrogen levels [39,40]. In Case 1, attenuation from the left breast reduced the apparent myocardial uptake, potentially causing a false-negative assessment [41]. In contrast, uptake in breast tissue or blood pool may mimic cardiac uptake, resulting in false-positive findings (Figure 1A,B). Other known causes of non-cardiac uptake that can lead to false-positive interpretations include hydroxychloroquine-associated cardiac toxicity, rib fractures, valvular or annular calcification, and recent myocardial infarction, underscoring the importance of thorough review of patient history and prior imaging [8]. Additionally, false-negative results can occur even when scintigraphy is negative; certain pathogenic TTR variants, such as Glu112Lys, are associated with amyloid deposits that do not show typical tracer uptake, requiring high clinical suspicion for diagnosis [42]. Therefore, careful attention and comparison with prior images are essential to avoid misdiagnosis (Figure 2). If further evaluation is warranted, reassessment with high-resolution static images and SPECT/CT should be considered, given the limited spatial resolution of whole-body bone scintigraphy (Figure 1) [43]. Beyond visual grading, SPECT/CT provides superior anatomical localization and precise assessment of myocardial tracer uptake.

In our cases, SPECT/CT was essential to exclude false-positive signals from the blood pool or overlapping rib uptake and to compensate for false-negative factors such as breast attenuation. As illustrated in Case 2 (Figure 1F), SPECT/CT revealed biventricular inferior and septal uptake, effectively ruling out potential rib uptake and providing a comprehensive characterization of disease distribution [44]. Furthermore, as emphasized by Wollenweber et al., tracer uptake in ATTR-CM is time-dependent, and delayed imaging provides more stable quantitative data and higher contrast [45]. In the present cases, SPECT/CT was acquired 3 h post-injection, consistent with these findings, thereby ensuring diagnostic reliability. These findings underscore the importance of recognizing both early and advanced presentations of ATTR-CM. In Case 2, the patient developed a cerebral infarction just prior to hospital admission for endomyocardial biopsy. The Perugini score was Grade 3 (greater than bone) (Figure 1D), indicating a more advanced stage of cardiac amyloid deposition at the time of diagnosis. Cardiac uptake was not evident on bone scintigraphy performed three years earlier; however, retrospective review suggests the possibility of equivocal uptake at that time, implying that amyloid deposition may have already been present (Figure 2C). Previous reports have shown that ischemic strokes may occur an average of 2.9 years after ATTR-CM diagnosis [32], underscoring the critical importance of early recognition and diagnosis. Taken together, recent advancements in quantitative SPECT/CT, such as DPD quantification as a novel biomarker [46], and the evaluation of regional variation—particularly right ventricular involvement [44]—further enhance its role in risk stratification. These emerging roles suggest that while planar bone scintigraphy remains a valuable screening tool in oncology patients, SPECT/CT with quantitative analysis should be strongly considered to improve both diagnostic specificity and prognostic stratification when ATTRwt-CM is suspected.

## 6. Thromboembolic Risk and Stroke in ATTR-CM: Clinical Implications and the Role of LAAC

Cardiac amyloidosis is associated with an increased risk of ischemic stroke, irrespective of AF [47,48]. In a retrospective cohort of ATTR-CM patients, 30.0% experienced cerebral ischemic events [32]. This is thought to result from intracardiac thrombus formation due to blood stasis, impaired wall motion from amyloid infiltration, endocardial damage, and a hypercoagulable state [48]. Ischemic stroke occurred as the initial manifestation of amyloidosis in approximately 30–40% of patients [32,49]. It was associated with poor prognosis, with a mean survival of 2.9 years after diagnosis [32]. In Case 2, the patient developed an ischemic stroke despite adequate anticoagulation and subsequently underwent LAAC. Recent reports on LAAC in ATTR-CM have shown high procedural success rates despite advanced age and multiple comorbidities. However, the incidence of stroke during a two-year follow-up was higher in the ATTR-CM group, suggesting that thrombi may originate not only from the left atrial appendage (LAA) but also from other sites. Nevertheless, LAA thrombus was detected preprocedure in 7.5% of ATTR-CM, compared to 2.9% of the control group, supporting the value of LAAC in this population. However, the need for continuation of anticoagulation therapy after LAAC remains controversial [50].

## 7. Current and Emerging Therapies for ATTR-CM

Transthyretin (TTR) is a tetrameric protein primarily synthesized in the liver. Dissociation of TTR tetramers into monomers can lead to protein misfolding and subsequent formation of amyloid fibrils, which are predominantly deposited in the heart, resulting in ATTR-CM [1]. Currently, therapeutic strategies for ATTR-CM can be categorized into three major approaches: (1) TTR stabilizers, which prevent tetramer dissociation; (2) TTR silencers (knockdown), which reduce hepatic synthesis of TTR; and (3) TTR depleters, which aim to clear existing amyloid fibrils.

TTR stabilizers inhibit tetramer dissociation, thereby preventing the cascade of misfolding and subsequent amyloid fibril formation. Tafamidis and acoramidis, both TTR stabilizers, have been shown to improve clinical outcomes, as demonstrated in the ATTR-ACT and ATTRibute-CM trials [51,52]. In patients aged ≥80 years, tafamidis therapy improved median survival (45 vs. 27 months) [53]. In early-stage patients (NYHA I–II), tafamidis reduced all-cause mortality by 44%, and even in NYHA III the reduction reached 35%, underscoring the benefit of initiating therapy before advanced disease progression [51]. Acoramidis is a TTR stabilizer that prevents tetramer dissociation and achieves over 90% ex vivo stabilization [54]. Treatment with acoramidis has also been shown to increase serum TTR, which reflects TTR stabilization. The naturally occurring T119M variant in the TTR gene enhances tetramer stability and has been associated with a lower incidence of cardiovascular events and prolonged survival [55].

TTR silencers, such as siRNA, antisense oligonucleotide (ASO), and emerging clustered regularly interspaced short palindromic repeats and associated Cas9 endonuclease (CRISPR-Cas9) gene-editing therapies, reduce hepatic TTR synthesis or expression by targeting the TTR gene or its mRNA. siRNA therapy with vutrisiran has demonstrated improved survival outcomes in the HELIOS-B trial [56,57]. CRISPR-Cas9–mediated therapies, such as NTLA-2001, are designed to provide a one-time treatment by permanently disrupting the TTR gene. NTLA-2001 contains mRNA encoding the Cas9 protein and a single guide RNA designed to knockout the TTR gene, both encapsulated within a lipid nanoparticle. In a first-in-human study, NTLA-2001 led to dose-dependent and durable reductions in serum TTR protein levels, with no clinically significant off-target editing or serious adverse events observed [58]. Notably, 92% of patients showed either improvement or no change in their NYHA class [59]. However, its long-term safety and efficacy remain to be fully established. The MAGNITUDE trial evaluates NTLA-2001 in transthyretin amyloid cardiomyopathy, with cardiovascular outcomes as the primary endpoint [9].

TTR depleter therapies promote antibody-mediated clearance by tagging amyloid deposits for removal by the immune system, primarily via macrophage phagocytosis. Recent TTR depleter therapies, including monoclonal antibodies such as NNC6019-0001, ALXN2220, and AT-02, have demonstrated early promise in reducing amyloid burden and improving cardiac and neurological outcomes in clinical and preclinical studies. These therapies represent a novel strategy that directly targets and eliminates existing amyloid deposits, complementing the effects of TTR stabilizers and silencers [9].

## 8. Management of Heart Failure in ATTR-CM

A combination of four pharmacologic agents—collectively termed the “Fantastic Four”—has become the standard of care for heart failure with reduced ejection fraction (EF) [60]. However, their role in the management of ATTR-CM remains controversial [61]. These agents include β-blockers, mineralocorticoid receptor antagonists (MRA), angiotensin receptor–neprilysin inhibitors (ARNI), and sodium–glucose cotransporter 2 inhibitors (SGLT2i). Recent studies have shown that MRA are effective regardless of EF, whereas β-blockers demonstrate efficacy only in patients with an EF ≤ 40%, and angiotensin receptor blockers (ARB) have shown no clear benefit, with high rates of treatment discontinuation. Although no data are available on the efficacy of ARNI in ATTR-CM, its use may be limited by the high discontinuation rate observed with ARBs, which is a component of ARNI. SGLT2i have also been reported to be effective; however, these studies included patients with a mean EF of approximately 45–50%, suggesting that the absolute treatment benefit may be greater in patients with mildly reduced EF [62,63].

Although no direct evidence supports the use of tolvaptan (a vasopressin V2 receptor antagonist) in ATTR-CM, it inhibits water reabsorption by reducing aquaporin (AQP)-2 expression in the renal collecting ducts, and it may contribute to loop diuretic dose reduction in clinical practice [61]. Wulingsan, known as Goreisan in Japan, is a traditional herbal formula used in both Chinese and Japanese medicine for managing heart failure. It has been shown to reduce AQP2 and AQP3 expression in the kidney without altering V2 receptor mRNA levels [64], suggesting a V2R-independent mechanism of action. In a real-world cohort, it was associated with reduced heart failure readmission in patients with renal disease [65]. Thus, tolvaptan and Goreisan may represent treatment options for fluid management in heart failure patients with renal dysfunction, although further studies are needed in ATTR-CM.

## 9. Early Diagnosis as a Catalyst for Social Impact: Improving Health Outcomes and Reducing Care Burden

Disease-modifying therapies offer the most significant clinical benefit when initiated at an early stage, and several assessments involving patients with NYHA class I–II symptoms revealed that tafamidis can reduce annual heart failure hospitalization by about four days [66]. Such reductions may help preserve quality of life (QoL) and potentially alleviate the financial burden associated with recurrent hospitalizations. In contrast, patients with NYHA class III symptoms require more intensive support than those in earlier stages, with increased caregiver burden in this population [67]. These findings indicate that early diagnosis may not only improve patient-level outcomes but also relieve caregiver strain and lessen broader socioeconomic consequences. Importantly, bone scintigraphy—routinely performed in elderly oncology populations—provides an exceptional opportunity for myocardial tracer uptake detection without requiring additional testing [5]. This incidental technique positions routine bone scintigraphy as a practical and cost-efficient tool for early identification of treatable ATTRwt-CM. Collectively, these findings emphasize the multidimensional advantages of early recognition of ATTRwt-CM, encompassing clinical, economic, and societal domains, and should be considered an integral component of modern disease management strategies.

## 10. Conclusions

While the existing literature reviews on incidental cardiac uptake in bone scintigraphy of cancer patients predominantly report male cases, the present study describes two female breast cancer patients who underwent early therapeutic intervention. These cases demonstrate the diagnostic pitfalls and the importance of serial imaging evaluation to assess temporal changes. The present cases and literature reviews underscore the clinical significance of incidental cardiac uptake observed during routine bone scintigraphy for metastatic evaluation, as such findings may suggest subclinical or asymptomatic ATTRwt-CM.

With advances in cancer therapies and increased life expectancy, the number of elderly cancer survivors at risk of ATTRwt-CM is expected to rise. Particularly in elderly cancer patients, incidental myocardial uptake should not be overlooked, and appropriate follow-up evaluation should be considered to facilitate early diagnosis and treatment of ATTRwt-CM. Early identification of myocardial uptake on routine bone scintigraphy may facilitate timely initiation of disease-modifying agents such as tafamidis or acoramidis, potentially improving clinical outcomes and QoL. In addition, incorporating myocardial evaluation into standard oncologic bone scintigraphy may provide a cost-effective strategy for the early detection of ATTRwt-CM in elderly cancer patients.

## Data Availability

No new data were created or analyzed in this study. Data sharing is not applicable to this article.

## Abbreviations

^99m^Tc-HMDP	Technetium-99m hydroxymethylene diphosphonate
ATTRwt-CM	Wild-type transthyretin cardiac amyloidosis
^99m^Tc-PYP	Technetium-99m pyrophosphate
^99m^Tc-DPD	Technetium-99m-3,3-diphosphono-1,2- propanodicarboxylic acid
ATTR-CM	Transthyretin amyloid cardiomyopathy
siRNA	Small interfering RNA
AF	Atrial fibrillation
hsTnT	High-sensitivity troponin T
NT-proBNP	N-terminal pro-brain natriuretic peptide
FLC	Free light chains
SPECT/CT	Single-photon emission computed tomography/computed tomography
LAAC	Left atrial appendage closure
^99m^Tc-MDP	Technetium-99m methylene diphosphonate
TTR	Transthyretin
ASO	Antisense oligonucleotide
CRISPR-Cas9	Clustered regularly interspaced short palindromic repeats and associated Cas9 endonuclease
H/CL	Heart-to-contralateral
EF	Ejection fraction
MRA	Mineralocorticoid receptor antagonists
ARNI	Angiotensin receptor–neprilysin inhibitors
ARB	Angiotensin receptor blockers
SGLT2i	Sodium–glucose cotransporter 2 inhibitors
AQP	Aquaporin
QoL	Quality of life
